# Effect of Synbiotic and Postbiotic Supplements on Dental Caries and Periodontal Diseases—A Comprehensive Review

**DOI:** 10.3390/ijerph22010072

**Published:** 2025-01-08

**Authors:** Svante Twetman, Daniel Belstrøm

**Affiliations:** Department of Odontology, Section for Clinical Oral Microbiology, Faculty of Health and Medical Sciences, University of Copenhagen, Nørre Alle’ 20, 2200 Copenhagen, Denmark; dbel@sund.ku.dk

**Keywords:** dental caries, dental biofilm, gingivitis, periodontitis, peri-implantitis

## Abstract

Caries and periodontitis affect a significant part of the global population. Regular oral hygiene, sugar restriction, and fluoride exposure are the main avenues for the maintenance of oral health, but the adjunctive use of prebiotics and probiotic bacteria has gained attention over the past decades. The microbial and clinical effects of these biological interventions have been thoroughly covered in systematic reviews. However, the combination of prebiotics and probiotics (synbiotics) may boost the clinical benefits, and postbiotics, being inanimate microorganisms, can, when added to oral hygiene products, offer a sustainable option. The aim of this narrative review was to summarize clinical trials on the adjunctive use of synbiotics and postbiotics in the prevention and management of dental caries, gingivitis, and periodontitis. We searched two databases (PubMed and Google Scholar) for relevant literature, and we identified 17 relevant papers, five on dental caries and 12 with periodontal endpoints. We found emerging evidence of low certainty that lozenges/tablets containing synbiotics or postbiotics could reduce caries incidence in preschool and schoolchildren in comparison with standard preventive care. The effect on adult patients with plaque-induced gingivitis was less consistent. For adults with periodontitis, the adjunctive use of synbiotic and postbiotic products seemed to enhance the outcome of conventional scaling and root planning. In conclusion, both dental caries and periodontitis are non-communicable diseases, closely associated with an unbalanced oral biofilm, and the application of microbial modulators, including synbiotics and postbiotics, display promising beneficial effects and warrant further research.

## 1. Introduction

Caries and periodontitis affect a significant part of the global population. According to current estimates, more than 2.8 billion children and adults have untreated caries, while 1.1 billion adults suffer from severe periodontitis and total tooth loss [1]. These high values are remarkable considering the preventable nature of these conditions. Caries and periodontitis belong to the group of non-communicable diseases with lifestyle and behavior-related causal factors, resulting in an unbalanced and dysbiotic oral biofilm [2,3]. Effective preventive strategies should therefore focus on re-establishing bacterial homeostasis rather than eradicating selected pathogens [4]. For this purpose, various biofilm-modulating strategies have gained interest over the past two decades, in particular prebiotics and probiotics. Probiotics are a group of live bacteria that confer a health benefit on the host when taken in sufficient amounts [5], whereas prebiotics are specific substrates that enhance the growth and activity of probiotic bacteria [6]. The impact of prebiotic and probiotic supplements on oral health has been covered in several systematic reviews in recent years [7,8,9,10]. The role of synbiotics and postbiotics in the prevention and management of oral diseases is however less explored. Synbiotics are a combination of prebiotics and probiotics [11], while the term postbiotics (sometimes called paraprobiotics) refers to inactivated probiotic bacteria, their metabolites, or extracted cell walls [12]. Synbiotics and postbiotics largely share the same mode of action with probiotics: promotion of homeostasis, exerting antimicrobial effects, and modulation of the immune response [13].

The effect of synbiotics and postbiotics on dental and oral biofilms has mainly been investigated in in vitro models [14]. For example, combinations of *Lactobacillus acidophilus* and fructo-oligosaccharides, and *Lactobacillus rhamnosus* and L-arginine, can inhibit the growth and biomass of *Streptococcus mutans* biofilms [4]. Heat-killed postbiotics from bifido- and lactic acid bacteria can reduce the cariogenicity of biofilms and co-aggregate with *S. mutans* [15]. Studies have also confirmed that postbiotics from lactic acid bacteria have the potential to inhibit pathogens related to periodontitis and peri-implantitis [16]. Examples are reduced colony-forming units of *Fusobacterium nucleatum* and *Porphymonas gingivalis*-induced periodontal injury [17], reduced integrity of biofilms, and suppressed gene expression in *P. gingivalis* biofilm formation [16]. Other demonstrated postbiotic effects include decreased levels of pro-inflammatory cytokines and suppression of RANK-L-mediated osteoclast differentiation [17,18]. To the best of our knowledge, no review paper has focused on the clinical effects of synbiotic and postbiotic supplements in controlled trials. The aim of this narrative review was therefore to summarize the effect of synbiotic and postbiotic supplements in the prevention and management of dental caries, gingivitis, and periodontitis as evaluated by data from human clinical trials.

## 2. Materials and Methods

We searched two databases (PubMed and Google Scholar) up to 15 October 2024 to retrieve the relevant literature. The main search terms in PubMed were as follows: (dental caries OR tooth decay OR childhood caries OR gingivitis OR gingival bleeding OR periodontitis OR periodontal disease) AND (synbiotic OR postbiotic OR parapostbiotic). We scanned the abstracts independently and included controlled human trials published in English with clinical endpoints related to dental caries, gingivitis, periodontitis, and peri-implantitis. When in doubt, the full text was reviewed. Studies in vitro, animal studies, and papers with only microbial and biochemical endpoints were discarded. In addition, we did not include articles dealing with oral hygiene interventions in hospitalized patients to prevent ventilator-associated pneumonia. We checked systematic and narrative reviews for additional potential references. We did not accept academic theses, non-peer-reviewed publications, grey literature, or commercial writings. One author extracted key data from the selected papers; this was double-checked and we summarized the main findings in heatmaps. We did not formally assess the risk of bias in the included papers, but we put most emphasis on results obtained from randomized placebo-controlled double-blinded trials. Due to heterogeneities in study designs, interventions, outcome measures, and follow-up protocols, it was not possible to conduct a meta-analysis. Therefore, we present the results as a narrative synthesis.

## 3. Results

We included 17 articles published between 2016 and 2024 in this review. We have summarized key data from the included studies in Table 1, along with the authors’ main conclusions.

### 3.1. Effect on Caries in Children

We identified five papers reporting on four trials in children and caries-active volunteers conducted in Denmark [21,22], Poland [20,23], and Mexico [19]. In three of them [20,21,22], caries was scored according to the International Caries Detection and Assessment System (ICDAS) [36] with 9–12 months follow-up. The largest trial evaluated the effect of daily synbiotic lozenges containing *L. rhamnosus*, *L. paracasei* and arginine on caries increment in primary and first permanent molars of young schoolchildren over 10–12 months, through clinical and radiographic scoring [21,22]. The children belonged to a low-caries population with access to preventive-oriented dental care, free of charge and the vast majority used fluoride toothpaste on a daily basis. The results showed a small but statistically significant reduction in the increment of moderate caries lesions in the mixed dentition in the test group compared with placebo controls [21]. The relative risk reduction for lesion progression (13.6%) was, however, not statistically significant [22]. Two studies from Poland evaluated the effect of postbiotics on caries incidence in preschool children [20,23]. After a very short intervention period (2 weeks), Staszczyk and colleagues [23] found significantly lower caries development after one year in the test group, compared with a “treatment as usual” control group. The second Polish study was a placebo-controlled trial on a group of caries-active preschool children [20]. The 9-month incidence of initial carious lesions was significantly lower in the active group compared with the placebo group. Notably, in the studies mentioned above [20,21,23], the level of oral hygiene and the occurrence of gingivitis were unaffected by the intervention. Likewise, the levels of salivary mutans streptococci and lactobacilli were not affected by the postbiotic intervention in one study [20]. We identified one trial with a surrogate endpoint for caries; a small study from Mexico demonstrated a slightly improved salivary buffer capacity among caries-active volunteers after a short-term intake of synbiotic capsules [19]. No product-related side effects were reported in any of the papers.

### 3.2. Effects on Gingivitis, Periodontitis and Peri-Implant Mucositis

We retrieved 12 papers with endpoints related to periodontal conditions, four utilizing synbiotics and eight postbiotics (Table 1). The countries of origin were China (n = 3) [26,27,28], Italy (n = 3) [30,31,35], India (n = 2) [33,34], Japan (n = 2) [24,32], Iran [29], and Turkey [25]. All but one [24] were conducted in adult patients with treatment-requiring disease, and the intervention was combined with and integrated into standard non-surgical procedures, such as oral hygiene control and scaling and root planing. The mode of intervention was local gel applications into periodontal pockets in three studies [31,34,35], active synbiotic capsules or tablets in five trials [24,25,29,32,33], and postbiotic toothpaste in four studies [26,27,28,30]. Common outcome measures were the amount of dental plaque (PI), gingival index (GI), bleeding on probing (BOP), pocket probing depth (PPD), and clinical attachment level (CAL). The follow-up periods ranged from four weeks to six months.

We show the detailed outcome according to the authors in Table 2, and a mixed and inconsistent pattern emerged. For patients with gingivitis, three studies reported significantly reduced values of PI and GI after 1–3 months of intervention when compared to baseline [24,25,28], but only one disclosed significant improvements in PI, BI, and BOP compared with a blank control [27]. For patients with periodontitis and peri-implantitis, the adjunctive use of synbiotics or postbiotics tended to be beneficial in terms of PPD in three studies when compared to the standard treatment. [30,32,33]. Likewise, improved CAL compared with the control groups was reported in three studies [29,30,33]. Four studies disclosed significant intragroup improvements in PPD [29,31,34,35] and CAL [31]. In addition, the clinical indices of gingival inflammation and dental plaque also seemed to be reduced by the various supplements among the periodontitis patients (Table 2). None of the publications described any adverse effects following the intervention.

## 4. Discussion

Dental caries, gingivitis, periodontitis, and peri-implantitis are all biofilm-mediated non-communicable diseases with several common risk factors, characterized by a dysbiotic abundance of aciduric, acidogenic, and/or proteolytic bacteria [2,3]. Prebiotics and probiotics, alone or in combinations, may help to restore dysbiotic oral ecosystems. Over recent decades, these microbiome-based supplements have gained attention for the prevention and care of oral diseases as an adjunct to conventional treatment. In this context, postbiotics may be of particular interest since they seem to retain the benefits of probiotics with better thermal stability, ease of storage, and longer shelf life [16,37]. In this review, we disclosed emerging but consistent evidence from three studies, of which two were conducted in high-caries populations, that both postbiotic and synbiotic supplements showed a small but significant decrease in caries incidence among young children. This was not a surprise since similar findings have been reported with probiotic supplements [38]. One explanation can be that the first years of life provide a window of opportunity to modulate the composition of the oral microbiota with “biotic” interventions [39,40]. The synbiotic study combined two probiotic strains, *Lacticaseibacillus rhamnosus* LGG and *Lactobacillus paracasei* subsp. *Paracasei*, with arginine [21,22], while the two postbiotic studies used tablets with heat-inactivated *Ligilactobacillus salivarius* CECT 5317 [20,23]. Two trials had robust placebo-controlled double-blind designs with sufficient duration and a validated capture of the caries scores [20,21]. It was, however, not possible to derive information on either dose-response relationships or a possible mechanism of action, as the trials did not exploit detailed analyses of the dental microbiome. It is anticipated that the prebiotic, probiotic, and postbiotic supplements can support dental biofilm balance by (i) co-aggregation with pathogens, (ii) competition for adhesion sites and nutrition, and (iii) bacteriocin production [4]. Interestingly, no beneficial effects on the amount of dental plaque or gingivitis were evident in the caries-related trials, and further studies on the mode of action seem warranted.

Based on the studies conducted on patients with gingivitis, we found mixed support for the supplements. Although the studies presented results pointing in the same direction, the improvements in gingival inflammation and bleeding were better than placebo in only one trial [27]. We found somewhat more consistent evidence that adjunctive use of synbiotics and postbiotics enhanced the effect of scaling and root planing in subjects with active periodontitis, as measured by clinically relevant endpoints (PPD and CAL) [29,30,33]. These findings are promising but must be verified in larger settings for several reasons. Firstly, standard non-surgical therapy is effective in reducing bleeding and periodontal pockets [41], and therefore, extended study populations are required to unveil the clinical benefit of adjunctive interventions. This was not the case in the present studies as half of them had a heterogeneous population in terms of age and critically low sample size. Secondly, many included papers reported short-term treatment effects, and only three studies had follow-up periods exceeding 12 weeks [30,31,34], and this was a clear limitation. Thirdly, some trials employed split-mouth [31,35] and crossover [26] designs, which is suboptimal due to the possible cross- and carry-over effect of active substances. Nevertheless, reduced values in probing pocket depth and improved clinical attachment level suggest that the adjunctive use of synbiotics and postbiotics may add patient value in comparison with scaling and root planning alone, but the evidence is not yet solid enough for clinical recommendations.

This observed ameliorating effect of synbiotics and postbiotics on the gingival tissues is in agreement with findings from in vitro and animal studies, demonstrating that certain pro-inflammatory mediators, for example, matrix metalloproteinase (MMP-8) and interleukins (IL-6), may be downregulated by the supplements [16,42,43]. We found some additional support in this review, as two of the retrieved papers reported significantly reduced concentrations of IL-6 and IL-10 in gingival crevicular fluid [25,34]. In four of the most recent periodontal studies, the use of postbiotic toothpaste was linked to simultaneous analyses of the dental biofilm [26,27,28,30]. Collectively, the findings indicated that postbiotic supplements could modulate oral micro-ecology and decrease the proportion of bacteria related to periodontal disease, often denoted as the “red complex” [30,44]. The use of postbiotic toothpaste was also associated with a shift, from dysbiosis to a more health-associated bacterial composition in patients with gingivitis [26]. However, effects on the alphadiversity of the plaque microbiota were inconsistent [27,45]. An overall impression was that postbiotic supplements derived from lactic acid bacteria seem to exert similar effects on periodontal diseases as live probiotic bacteria [10,46], thereby matching the effect of antibiotics [47]. None of the studies reported adverse effects due to the interventions, which was expected considering the safe nature of synbiotics and postbiotics. On the contrary, one finding was that postbiotic toothpaste mitigated adverse effects of sodium lauryl sulfate when used together [28].

The emerging evidence for the “biotics” in promoting oral health motivates further clinical research in all age groups. Dose-response effects, best mode of administration, optimal bacterial strains, and suitable combinations of prebiotics probiotics are still open questions. In addition, the postbiotics evaluated so far are based on heat-inactivated bacteria or cell-free suspensions, but there are more technologies available, such as ultrasonication, magnetic technologies, and plasma technologies [4]. Another knowledge gap is the lack of studies conducted on frail elderly and their dental microbiome. This age group constitutes an increasing part of the population, in which cognitive impairment may jeopardize their regular oral hygiene procedures, accelerate root caries activity, and increase tooth loss. Furthermore, health-economic calculations for the various modes of distribution and application are also lacking, albeit self-applied toothpaste may appear cost-effective, provided the product is affordable for consumers. We argue that an internationally agreed core outcome set for the reporting of clinical trials would be desirable to facilitate future meta-analysis. Future clinical studies should include biochemical analyses and biofilm samples for DNA-sequencing and bioinformatics for expanded knowledge of the mechanisms of action. Furthermore, since the oral/dental microbiome is unique for each individual, a future challenge is to tailor personalized supplements for optimal impact.

## 5. Conclusions

From the present literature, we found consistent evidence of low certainty that tablets or lozenges containing synbiotics or postbiotics may reduce caries incidence and progression in preschool and young schoolchildren. We also noted a tendency for daily use of postbiotic toothpaste to reduce gingival inflammation in patients with plaque-induced gingivitis, but the evidence was very uncertain. For adults with periodontal disease, the adjunctive intake of synbiotic or postbiotic tablets or capsules, as well as topical applications, seemed to enhance the outcome of conventional scaling and root planning. No product-related side effects were reported. Further clinical studies are required to confirm or refute the supplementary contribution of synbiotics and postbiotics in the context of caries and periodontal disease.

## Figures and Tables

**Table 1:** Available studies on synbiotics (light blue) and postbiotics (gray) on dental caries, gingivitis, periodontitis, and peri-implantitis. Significant beneficial findings are marked in light green; possibly beneficial findings are marked in yellow.

First Author, Year; Country [Reference]	Age, Sample Size, Duration	Intervention	Placebo/Control	Author’s Reported Result
**Dental caries**				
Hernandez 2020; Mexico [19]	NR, 24 volunteers with active caries; 6 d.	Synbiotic capsules; *Lactobacillus* + Vitamin B complex	None	Salivary buffer capacity was short-time improved
Olczak-Kowalczyk, 2024; Poland [20]	Caries-active preschool children; n = 73; 9 mo. follow-up	Postbiotic tablets; inactivated *L. salivarius* + cranberry extract	Placebo tablets	Significantly reduced incidence of initial carious lesions
Pørksen, 2023; Denmark [21,22]	5–9 years, n = 343; 3 mo. 10–12 mo. follow-up	Synbiotic lozenges; *L. rhamnosus* + *L. paracasei* + arginine	Placebo Lozenges	Significantly reduced caries increment in the test group; no effects on plaque amount or occurrence of gingivitis
Staszczyk, 2022; Poland [23]	3–6 years, n = 140 2 w. 1 yr follow-up	Postbiotic tablets; inactivated *L. salivarius* + xylitol	Treatment as usual	Significantly lower caries incidence in the test group; no effect on oral hygiene
**Dental plaque**				
Tobita, 2018; Japan [24] Healthy subjects	NR, n = 16 4 w.	Daily postbiotic tablet with heat-killed *L. crispatus*	Placebo tablet, once daily	Decreased plaque scores
**Gingivitis**				
Ercan, 2020; Turkey [25]	18–30 years, n = 80; 2 mo. follow-up	Synbiotic tablet daily 6 probiotic strains + fructooligosaccharides	Placebo tablet Daily	No significant differences in GI and PI between the groups
Lee, 2024: China [26]	20–59 years, n = 30; 4 w.	Cross-over trial; postbiotic toothpaste with heat-killed *L. paracasei*	Cross-over trial; Placebo toothpaste	Significantly reduced GI, but not PI, compared with the control
Li, 2024; China [27]	18–25 years, n = 60; 3 mo.	Postbiotic toothpaste with inactivated *L. paracasei*	Blank toothpaste	Significantly reduced BOP, GI, and PI compared with the control
Shi, 2024; China [28]	NR, n = 144; 30 d.	Postbiotic toothpaste (20% lactobacilli fermentation extract)	Postbiotic toothpaste + SLS	No significant differences between the groups
**Periodontitis**				
Bazyar, 2020 Iran [29]	n = 47 with diabetes; 8 w.	Synbiotic capsule; 7 probiotic strains + fructooligosaccharides + SRP	Placebo capsule + SRP	Significant reduction of CAL and bleeding on probing in the test group
Butera, 2022; Italy [30]	18–70 years, n = 40 6 mo. follow-up	Postbiotic toothpaste and mouthwash (Biorepair) + SRP	CHX toothpaste (0.2%) + SRP	Significantly reduced BOP, PPD, and CAL compared with the control
Butera, 2022; Italy [31]	18–70 years, n = 20 6 mo. follow-up	Split-mouth; 4 × intrapocket postbiotic gel (Biorepair) + SRP	Split-mouth; 4 × 1% CHX gel + SRP	Significant decrease in PPD, PI, and GBI in both groups
Iwasaki, 2016; Japan [32]	NR, n = 39; 12 w.	Daily postbiotic capsule with heat-killed *L. plantarum* + SRP	Placebo capsule daily + SRP	BOP and PPD significantly reduced compared with the control
Murugesan, 2018; India [33]	18–30 years; n = 60; 8 w.	Synbiotic lozenge (not specified) + SRP	Placebo lozenge + SRP	Significantly reduced PPD, CAL, and BOP compared with the control
Shetty, 2020 India [34]	25–45 years, n = 180 6 mo. follow-up	4 × intrapocket synbiotic gel (BIFILKACR), combined with SRP	SRP only	No differences in GI, PI, or PPD between groups
**Peri-implantitis**				
Butera, 2022 Italy [35]	18–70 years; n = 30 6 mo. follow-up	Split-mouth; 2 × Postbiotic gel (Biorepair intensive)	Split-mouth; 2 × 1% CHX gel	Significantly reduced GBI, but not PI and PPD compared with the control group

Abbreviations: BOP = bleeding on probing; CAL = clinical attachment level; CHX = chlorhexidine; GBI = gingival bleeding index; GI = gingival index; NR= not reported; PI = plaque index; PPD = pocket probing depth; SLS = sodium lauryl sulfate; SRP = scaling and root planing.

**Table 2:** Effect of synbiotic and postbiotic interventions on periodontal health. Light green ⊕ indicates a statistically significant reduction/improvement compared with the control group (intergroup comparison); yellow ⊖ indicates a statistically significant reduction/improvement compared with baseline (intragroup comparison).

First Author, Year [Ref]	Condition	SRP	PI	GI	GBI/BOP	PPD	CAL
Tobita, 2018 [24]	Healthy patients	No	⊕				
Ercan, 2020 [25]	Gingivitis	No	⊖	⊖			
Lee, 2024 [26]	Gingivitis	No	⊖	⊖			
Li, 2024 [27]	Gingivitis	No	⊕	⊕	⊕		
Shi, 2024 [28]	Gingivitis	No	⊖	⊖			
Bazyar, 2020 [29]	Periodontitis	Yes	⊕		⊖	⊖	⊕
Butera, 2022 [30]	Periodontitis	Yes	⊖		⊕	⊕	⊕
Butera, 2022 [31]	Periodontitis	Yes	⊖		⊖	⊖	⊖
Iwasaki, 2016 [32]	Periodontitis	Yes			⊖	⊕	
Murugesan, 2018 [33]	Aggressive periodontitis	Yes	⊖		⊕	⊕	⊕
Shetty, 2020 [34]	Periodontitis	Yes	⊖	⊖		⊖	
Butera, 2022 [35]	Peri-implant mucositis	Yes	⊖		⊕	⊖	

Abbreviations: SRP = scaling and root planing/non-surgical supportive therapy; PI = plaque index; GI = gingival index; GBI/BOP = gingival bleeding index/bleeding on probing; PPD = pocket probing depth; CAL = clinical attachment level.

## Data Availability

No new data were created or analyzed in this study. Data sharing is not applicable to this article.
